# 1740. Healthcare Providers’ Preferences for Pediatric Pneumococcal Vaccination Recommendations in the United States

**DOI:** 10.1093/ofid/ofad500.1571

**Published:** 2023-11-27

**Authors:** Jeffrey T Vietri, Kelley H Myers, Liping Huang, Vincenza Snow, Brett Hauber, Adriano Arguedas, Alejandro D Cane, Maria J Tort, Anna Pierce, Christine Poulos

**Affiliations:** Pfizer, Inc., Collegeville, Pennsylvania; RTI Health Solutions, Garnet Valley, Pennsylvania; Pfizer Inc, Collegeville, Pennsylvania; Pfizer Vaccines, Collegeville, PA; Pfizer, Inc., Collegeville, Pennsylvania; Pfizer, Collegeville, Pennsylvania; Pfizer, Collegeville, Pennsylvania; Pfizer Inc, Collegeville, Pennsylvania; RTI Health Solutions, Garnet Valley, Pennsylvania; RTI-Health Solutions, Research Triangle Park, NC

## Abstract

**Background:**

A 20-valent pneumococcal conjugate vaccine (PCV20) was recently licensed for use in children by the United States (US) Food and Drug Administration. PCV20 includes 5 and 7 more serotypes than the 15- (PCV15) and 13-valent (PCV13) vaccines, respectively. This study assessed healthcare providers’ (HCPs) preferences for possible changes in pediatric pneumococcal vaccination recommendations, including transitioning from lower-valent vaccines to PCV20 among children partially vaccinated with PCV13/15, administering a supplemental dose of PCV20 among children < 5 years vaccinated with PCV13/15, and using PCV20 alone instead of in sequence with 23-valent pneumococcal polysaccharide vaccine (PPSV23) in children with underlying medical conditions. HCP preferences for the use of PCV15 or PCV20 for children aged ≤ 18 years were also elicited.

**Methods:**

Pediatricians (N = 268), family medicine physicians/general practitioners (N = 184), physician assistants (N = 77), and nurse practitioners (N = 74) who prescribed, recommended, or administered a pneumococcal vaccine to a child ≤ 18 years in the past 3 months completed an online survey. HCPs responded to direct elicitation questions to assess preferences for each of the potential pneumococcal vaccination recommendations considered above. Practice characteristics, knowledge and awareness of current pediatric pneumococcal vaccination recommendations, and rationale for choices in the preference questions were also assessed.

**Results:**

HCPs preferred recommendations that provided protection against more serotypes; 76% of HCPs in this study favored transitioning children who started vaccination with PCV13/15 to PCV20 (Figure 1A), and most (≥ 60%) favored a supplemental dose of PCV20 for all children < 5 years fully vaccinated with PCV13/15 (Figure 1B/1C). 93% of surveyed HCPs preferred PCV20 over PCV15 for children ≤ 18 years (Figure 1E). Among those who preferred PCV20, more than 80% based their decision mainly on serotype coverage.

**Figure 1.**
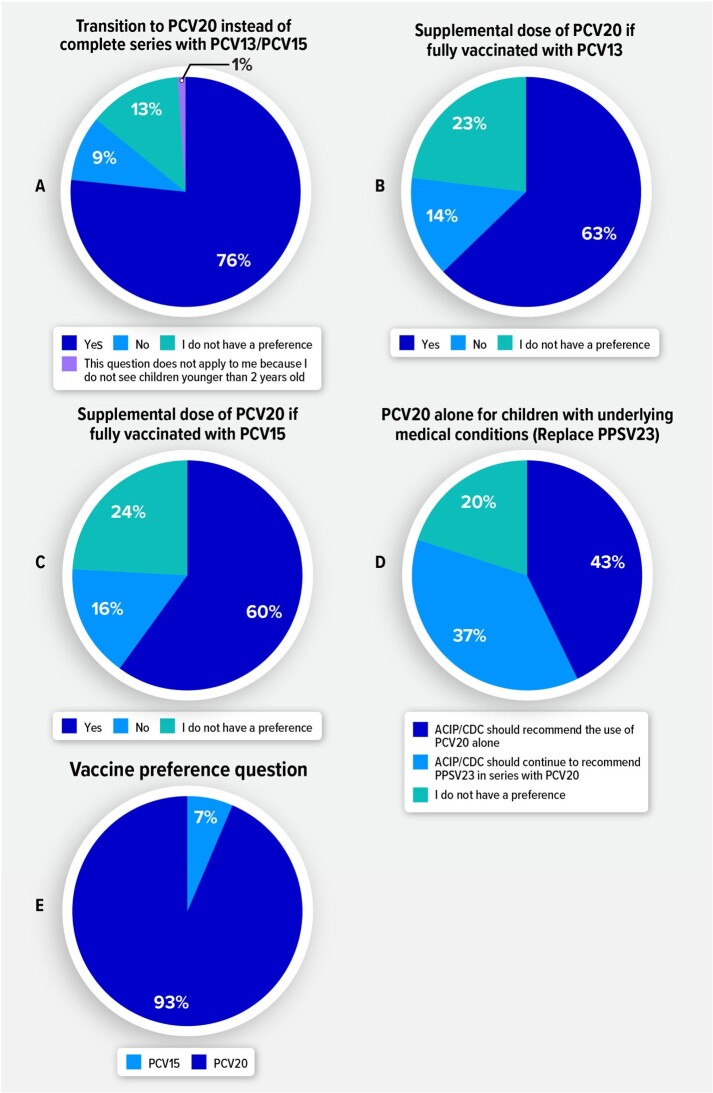
HCP Preferences for Potential Changes to Current Pediatric Pneumococcal Vaccine Recommendations

**Conclusion:**

HCPs indicated preference for pediatric pneumococcal vaccination recommendations that provide greater serotype coverage. These results and other data on stakeholder preferences will help inform the updated pediatric pneumococcal vaccination recommendations.

**Disclosures:**

**Jeffrey T. Vietri, PhD**, Pfizer Inc: Employment|Pfizer Inc: Stocks/Bonds **Liping Huang, MD, MA, MS**, Pfizer Inc.: Stocks/Bonds **Vincenza Snow, MD**, Pfizer Vaccines: employee|Pfizer Vaccines: Stocks/Bonds **Brett Hauber, PhD**, Pfizer: Brett Hauber is an employee of Pfizer. this study was funded by Pfizer, Inc.|Pfizer: Stocks/Bonds **Adriano Arguedas, Medical director**, Pfizer: Emplyee|Pfizer: Stocks/Bonds **Alejandro D. Cane, MD, PhD**, Pfizer: Stocks/Bonds **Maria J Tort, PhD**, Pfizer Inc: Stocks/Bonds **Anna Pierce, BBA**, Pfizer, Inc.: Grant/Research Support

